# Checkpoint inhibitor–induced hematologic immune-related adverse events and their association with disease recurrence in hematolymphoid malignancies: a clinical perspective

**DOI:** 10.1007/s44313-026-00135-4

**Published:** 2026-05-06

**Authors:** Chokri Ben Lamine, Amr Hanbali, Ghada ElGohary, Hanan Alkhaldi, Riad El Fakih, Mahmoud Aljurf

**Affiliations:** 1 Department of Hematology, Stem Cell Transplantation and Cellular Therapy, King Faisal Specialist Hospital and Research Centre, Cancer Centre of Excellence, Riyadh, Saudi Arabia; 2 Alfaisal University, Riyadh, Saudi Arabia; 3 Internal Medicine Department/Hematology Center, Ain Shams University, Cairo, Egypt; 4 Adult Hematology Consultant, SMC Hospitals, Riyadh, Saudi Arabia

**Keywords:** Check point inhibitor, Hematological, Outcome, Toxicities, Auto-immune, Cytopenia

## Abstract

Immune checkpoint inhibitors have transformed the treatment of hematolymphoid malignancies by enhancing antitumor immunity. These agents are associated with immune-related adverse events (irAEs), including hematologic toxicities such as autoimmune hemolytic anemia, immune-related thrombocytopenia (irTCP), and neutropenia. Although rare, hematologic irAEs (hem-irAEs) may reflect systemic immune activation and could serve as biomarkers of treatment response. Emerging evidence, particularly from observational studies and meta-analyses, suggests that patients who experience hem-irAEs may have lower rates of disease recurrence and improved survival outcomes. This manuscript reviews the mechanisms, incidence, and prognostic implications of hem-irAEs in patients with hematolymphoid malignancies, emphasizing their hypothesis-generating potential as predictive markers that warrant prospective validation.

## Introduction

Hematolymphoid malignancies are a heterogeneous group of tumors originating from hematopoietic and lymphoid tissues. The incidence of hematologic and lymphoid cancers has increased significantly in recent years, emphasizing the need for novel and effective therapeutic strategies [1]. Despite advances in treatment and the development of new regimens, achieving durable remission remains challenging. Traditional therapies include chemotherapy, radiotherapy, and stem cell transplantation [2, 3]. Immunotherapy, including immune checkpoint inhibitors (ICIs), has emerged as a promising therapeutic strategy to overcome treatment resistance [4]. ICIs, including anti–CTLA-4 and anti–PD-1/PD-L1 agents, have revolutionized the landscape of cancer treatment, demonstrating remarkable efficacy across a wide range of malignancies, including Hodgkin lymphoma (HL) and other hematologic cancers [5–8]. These agents function by unleashing the body’s inherent immune response against cancer cells, primarily by blocking inhibitory pathways that suppress T-cell activity [9]. By reversing T-cell exhaustion and promoting sustained cytotoxic responses, ICIs can induce durable remissions and significantly improve patient outcomes [10, 11]. However, this potent immune activation is not without drawbacks. A major challenge associated with ICI therapy is the emergence of immune-related adverse events (irAEs), which are inflammatory conditions that affect various organs and tissues. Among these, hematologic manifestations, although relatively uncommon, are particularly noteworthy because of their potential severity and distinct clinical implications [12]. These include autoimmune hemolytic anemia (AIHA), immune-related thrombocytopenia (irTCP), neutropenia, aplastic anemia, and hemophagocytic lymphohistiocytosis (HLH) [13]. Although these toxicities pose significant management challenges, a growing body of evidence suggests a paradoxical association between their development and enhanced antitumor responses [14]. This observation has led to the hypothesis that hematologic irAEs (hem-irAEs) may serve as surrogate biomarkers of more robust and effective systemic immune activation against malignancy, potentially correlating with a lower risk of disease recurrence and improved survival outcomes [15]. This study aims to provide a comprehensive review of the current understanding of hem-irAEs in the context of hematolymphoid malignancies and to explore their underlying mechanisms, reported incidence, and prognostic significance as potential predictive markers of therapeutic efficacy.

## Mechanisms of hem-irAEs

Several mechanisms are thought to contribute to the pathogenesis of hem-irAEs.

- CD8 + T-cell–mediated cytotoxicity: involves direct cytotoxic effects exerted by activated CD8 + T cells. Although these T cells are crucial for targeting and eliminating malignant cells, their enhanced activity may result in cross-reactivity with normal hematopoietic progenitors. This phenomenon occurs when tumor-associated antigens share molecular homology with antigens expressed on healthy hematopoietic cells, leading to a misdirected immune response against bone marrow cells [16, 17]. This T-cell–mediated destruction may result in conditions such as aplastic anemia or pure red cell aplasia.
- Dysregulation of B-cell function and autoantibody production: ICI therapy can disrupt B-cell homeostasis, leading to the production of autoantibodies. These autoantibodies may target various blood cell components, such as red blood cell antigens, resulting in AIHA, or platelet glycoproteins, leading to irTCP [18, 19]. This dysregulation may involve a breakdown of B-cell tolerance checkpoints, allowing self-reactive B-cell clones to escape immune control and produce pathogenic autoantibodies.
- Hyperactivation of macrophages and natural killer (NK) cells: in severe cases, such as HLH, immune dysregulation extends to excessive activation of macrophages and NK cells, leading to uncontrolled inflammation and a cytokine storm. This process may cause multiorgan damage, including bone marrow suppression and pancytopenia [20].
- Cytokine release syndrome: the widespread immune activation induced by ICIs may lead to excessive release of proinflammatory cytokines. Although not a direct mechanism of cellular destruction, this cytokine milieu may contribute to systemic inflammation and exacerbate damage to hematopoietic cells [21, 22].

Collectively, these mechanisms reflect broader systemic immune stimulation. The hypothesis that hem-irAEs may represent a surrogate marker of therapeutic efficacy stems from the observation that patients who develop these adverse events often exhibit a more robust and widespread antitumor immune response.

## Incidence of hem-irAEs in hematolymphoid malignancies

Although most data on irAEs are derived from studies in solid tumors, the incidence and clinical characteristics of hem-irAEs in patients with hematolymphoid malignancies are increasingly being recognized. Overall, hem-irAEs are uncommon, with an estimated incidence of less than 3% among all patients treated with ICIs [23, 24]. However, this rate may vary depending on the specific ICI used, the type of underlying malignancy, and patient-related factors. Among the various hem-irAEs, AIHA, irTCP, and neutropenia are the most frequently reported complications. Together, these three conditions account for approximately 75–80% of all reported hem-irAEs. More specifically, AIHA and neutropenia each represent approximately 26% of hem-irAEs, whereas irTCP is slightly more common (approximately 29%). Less frequent but often more severe hem-irAEs, such as HLH and aplastic anemia, constitute the remaining proportion, with an estimated incidence of less than 0.5% among all ICI-treated patients [13, 15, 25, 26]. Notably, the true incidence of hem-irAEs in hematolymphoid malignancies is likely underreported and underestimated. Diagnosis may be challenging, as cytopenias are common in this patient population because of multiple factors, including the underlying disease, prior chemotherapy, and infections. A high index of suspicion and careful exclusion of alternative etiologies are essential for accurate diagnosis and reporting Tables 1 and 2.

**Table 1:** Incidence of hematologic irAEs in hematolymphoid malignancies

Hem-irAE type	Approximate proportion of Hem-irAEs	Estimated incidence among all ICI-treated patients
Autoimmune Hemolytic Anemia (AIHA)	~26%	<1%
Immune-Related Thrombocytopenia (irTCP)	~29%	~1%
Neutropenia	~26%	<1%
HLH/Aplastic Anemia (including marrow failure)	~19%	<0.5%

**Table 2:** Hem-irAEs and recurrence-free survival in selected studies

Study	Population	Hem-irAE type	Outcome	Key findings
Delanoy et al. (2019) [13]	Mixed solid and hematologic tumors	irTCP/AIHA	OS	Longer OS observed in patients with hem-irAEs
Tanios et al. (2019) [26]	FDA adverse event reporting system database	AIHA	Anecdotal RFS	Prolonged remission reported in some lymphoma cases
Ohashi et al. (2021) [15]	Meta-analysis (more than 9,000 patients)	Anemia/TCP	OS/PFS	Improved survival outcomes associated with hem-irAEs
Kramer et al. (2021) [27]	Multicenter study (18 centers; 7,626 ICI-treated patients)	All hem-irAEs	Incidence and outcomes	0.6% incidence; 78% resolution; clinical course characterized
Hradska et al. (2021) [28]	Prospective blood cancer trials (ICIs)	All irAEs	Toxicity profile	Characterized irAE incidence specifically in hematological malignancies

**Figure:**
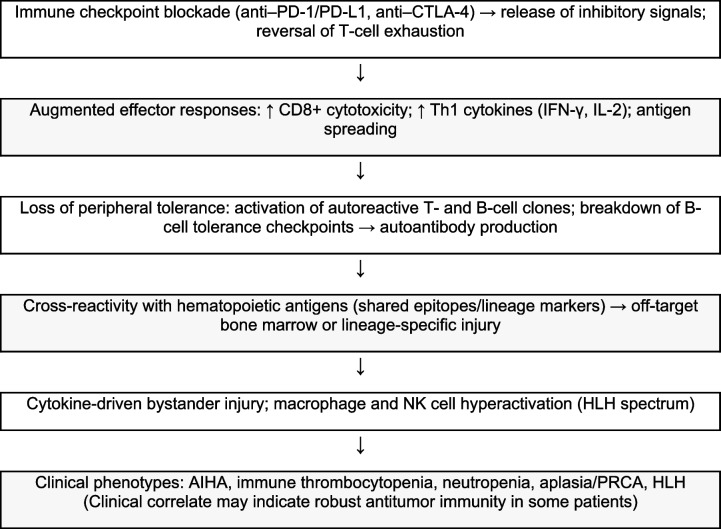
**Algorithm 1** Mechanism of checkpoint inhibitor–induced hematologic irAEs. Abbreviations: AIHA, autoimmune hemolytic anemia; ANC, absolute neutrophil count; CD8⁺, cluster of differentiation 8–positive cytotoxic T lymphocytes; CTLA-4, cytotoxic T-lymphocyte–associated protein 4; HLH, hemophagocytic lymphohistiocytosis; IFN-γ, interferon gamma; IL-2, interleukin-2; irAEs, immune-related adverse events; NK cells, natural killer cells; PD-1, programmed cell death protein 1; PD-L1, programmed death-ligand 1; PRCA, pure red cell aplasia; Th1, T helper type 1

**Figure:**
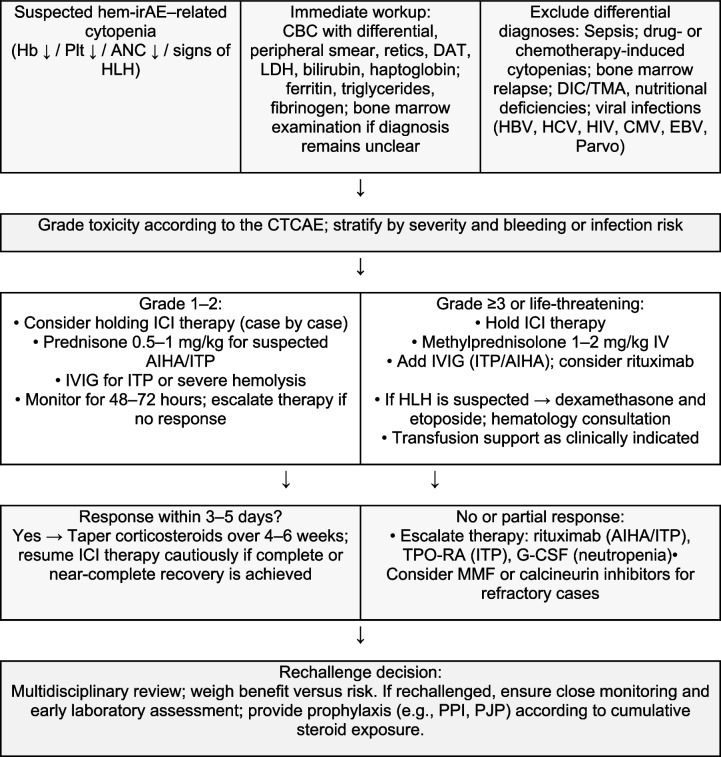
**Algorithm 2** Clinical management strategy for hematologic irAEs. Abbreviations: AIHA, autoimmune hemolytic anemia; ANC, absolute neutrophil count; CBC, complete blood count; CMV, cytomegalovirus; CTCAE, Common Terminology Criteria for Adverse Events; DAT, direct antiglobulin test; DIC, disseminated intravascular coagulation; EBV, Epstein–Barr virus; G-CSF, granulocyte colony–stimulating factor; Hb, hemoglobin; HBV, hepatitis B virus; HCV, hepatitis C virus; HIV, human immunodeficiency virus; HLH, hemophagocytic lymphohistiocytosis; ICI, immune checkpoint inhibitor; ITP, immune thrombocytopenia; IVIG, intravenous immunoglobulin; LDH, lactate dehydrogenase; MMF, mycophenolate mofetil; PJP, Pneumocystis jirovecii pneumonia; Plt, platelet count; PPI, proton pump inhibitor; TMA, thrombotic microangiopathy; TPO-RA, thrombopoietin receptor agonist

**Figure:**
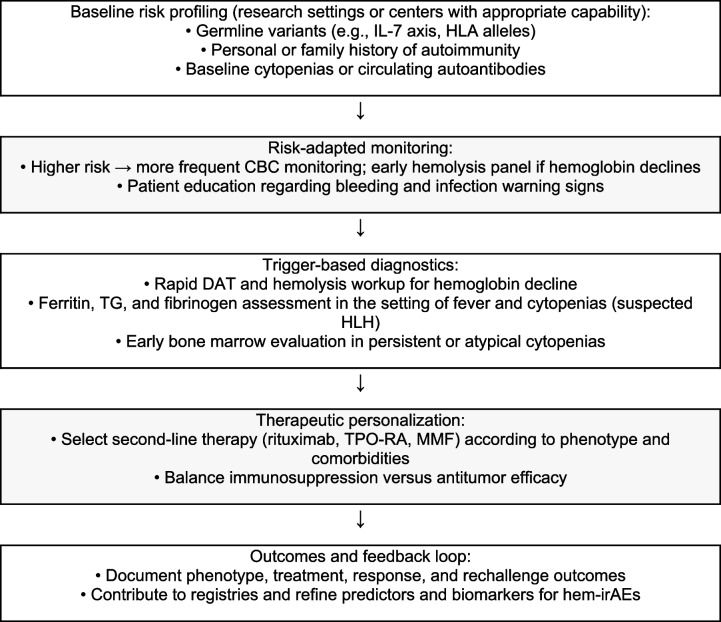
**Algorithm 3** Role of genetic predisposition and personalization in hematologic irAEs. Abbreviations: CBC, complete blood count; DAT, direct antiglobulin test; HLA, human leukocyte antigen; HLH, hemophagocytic lymphohistiocytosis; IL-7, interleukin-7; irAEs, immune-related adverse events; MMF, mycophenolate mofetil; TG, triglycerides; TPO-RA, thrombopoietin receptor agonist

## Association between hem-irAEs and disease recurrence

Several findings support a potential association between improved disease control and the occurrence of hem-irAEs. However, most of the existing evidence is derived from mixed populations of patients with solid tumors and hematologic malignancies, with limited data specific to hematolymphoid malignancies. Delanoy et al. [13] reported improved overall survival (OS) among patients who developed hem-irAEs. Although their cohort included a mixed population of solid tumors and hematologic malignancies, the findings suggest that immune activation leading to irAEs correlates with improved clinical outcomes [13]. A review by Tanios et al. (2019) of Food and Drug Administration (FDA) adverse event data included 68 cases of AIHA, some occurring in patients with lymphoma, and demonstrated prolonged remission in those who developed AIHA [26]. These case-based observations, while not definitive, provide preliminary evidence of an association between hem-irAEs and sustained disease control. A large meta-analysis by Ohashi et al. [15], including more than 9,000 patients, found a significant association between the occurrence of hematologic toxicities, specifically anemia and thrombocytopenia, after ICI therapy and improved OS and Progression-free survival [15]. This meta-analysis provides stronger statistical support for the hypothesis that hem-irAEs may have prognostic value, suggesting that these events are not merely adverse effects but may represent indicators of a robust antitumor immune response. Additionally, a multicenter retrospective study by Kramer et al. [27], which analyzed 50 patients with hem-irAEs across 18 international cancer centers, reported an overall incidence of 0.6% and documented outcomes including event resolution in 78% of cases, further characterizing the clinical course of these events. Hrádská et al. [28] reviewed prospective clinical trials in blood cancers investigating ICIs with available toxicity data, providing important insights into the incidence of irAEs specifically in the hematologic malignancy setting. Collectively, these findings are hypothesis-generating and suggest a potential association between hem-irAEs and favorable outcomes; however, prospective validation remains essential. Although mild to moderate irAEs are often associated with improved outcomes, severe or life-threatening irAEs may necessitate treatment interruption or immunosuppressive therapy, which could potentially attenuate the antitumor response. Therefore, careful management of irAEs is essential to maximize the therapeutic benefits of ICI therapy while minimizing adverse effects.

## Discussion

The observed association between hem-irAEs and favorable clinical outcomes in patients treated with ICIs represents a compelling area of ongoing investigation. This relationship suggests that hem-irAEs may serve as indicators of a robust and effective antitumor response. The underlying premise is that ICIs elicit widespread immune activation, and individuals who develop hem-irAEs may be those whose immune systems are particularly primed to mount broader T-cell responses capable of effectively targeting tumor antigens [29]. It is important to acknowledge, however, that much of the evidence supporting this association originates from studies in solid tumors or mixed populations, with HL being the only hematolymphoid malignancy for which ICI data are relatively well established. Extrapolation of these findings to the broader spectrum of hematolymphoid malignancies should therefore be undertaken with caution.

One of the key hypotheses explaining this dual effect—both adverse event and prognostic indicator—is immune cross-reactivity. In hematolymphoid malignancies, malignant cells often arise from the hematopoietic lineage and share lineage markers with their normal counterparts. This similarity may result in activated immune cells, particularly T cells, recognizing and attacking both malignant and healthy hematopoietic cells. This collateral damage, manifesting as hem-irAEs, may therefore represent an unintended consequence of a highly effective antitumor immune response [26, 30]. The immune system, when hyperstimulated, may not perfectly distinguish between malignant and healthy cells that present similar antigenic profiles. Recent advances in genetic research have shed light on the role of genetic predisposition in the development of irAEs. Studies suggest that certain germline variants, such as those within the interleukin-7 (IL-7) pathway, are associated with increased susceptibility to irAEs and, notably, with improved therapeutic responses to ICI therapy [31]. Identifying these genetic markers may facilitate personalized treatment strategies, whereby patients at higher risk of irAEs—but also with a greater likelihood of response—can be monitored more closely or managed proactively. From a clinical perspective, these insights are highly significant. The onset of hem-irAEs may carry prognostic value and may assist clinicians in assessing the likelihood of a favorable response to ICI therapy. However, this issue must be approached with caution. The management of irAEs often involves immunosuppressive therapies, such as corticosteroids, which may attenuate the very antitumor immunity being harnessed. The optimal timing, duration, and intensity of immunosuppression, as well as decisions regarding the continuation or discontinuation of ICI therapy during an irAE, remain critical clinical challenges that require further investigation [32]. Balancing the need to control severe irAEs with the goal of maintaining antitumor efficacy is a delicate undertaking. In summary, the discussion surrounding hem-irAEs extends far beyond their classification as treatment-related toxicities. These events represent an intersection of immune activation, breakdown of self-tolerance, and antitumor efficacy. Understanding these complex relationships is essential for refining patient selection, optimizing treatment strategies, and ultimately improving outcomes for patients with hematolymphoid malignancies treated with ICIs.

## Limitations and future directions

Despite the reported findings regarding the prognostic implications of hem-irAEs in patients treated with ICIs, several limitations in the current evidence base warrant further investigation. Notably, the available data are predominantly derived from studies in solid tumors or mixed populations, with hematolymphoid malignancy–specific data remaining limited. Addressing these limitations is essential to achieve a more comprehensive understanding and to facilitate translation of these insights into clinical practice.

Current limitations:

- Retrospective design and limited sample sizes: The existing data on hem-irAEs are primarily derived from retrospective studies and case series. These study designs are subject to inherent biases, limited sample sizes, and lack of standardized reporting, all of which may affect the generalizability and robustness of the findings. However, the rarity of certain hem-irAEs limits the feasibility of conducting large-scale prospective studies.
- Inconsistent definitions and diagnostic criteria: Definitions and diagnostic criteria for hem-irAEs vary across studies, leading to inconsistencies in reported incidence rates and complicating direct comparisons. This heterogeneity may obscure the true prevalence and clinical characteristics of these events.
- Confounding factors: Distinguishing hem-irAEs from other causes of cytopenias (e.g., infection, disease progression, prior chemotherapy, or other drug-related toxicities) in patients with hematolymphoid malignancies is often challenging. This diagnostic complexity makes it difficult to attribute cytopenias definitively to ICI therapy and may affect the accuracy of reported associations.
- Lack of prospective validation: Although retrospective data suggest an association between hem-irAEs and improved outcomes, prospective, controlled trials are lacking. Such studies are necessary to confirm the observed associations, establish causality, and accurately quantify the prognostic value of hem-irAEs.

### Future directions

To address these limitations and advance understanding of hem-irAEs, several key areas of future research warrant attention:

- Prospective registries and standardized documentation: Establishing large prospective registries with standardized protocols for irAE documentation—including detailed clinical characteristics, laboratory parameters, and treatment outcomes—is essential. Such initiatives would promote consistent data collection and facilitate robust analyses.
- Biomarker studies: Comprehensive biomarker investigations are needed to elucidate the immunologic mechanisms underlying hem-irAEs. This includes assessing autoantibody profiles, characterizing T-cell and B-cell subsets (e.g., T-cell receptor clonality and B-cell repertoire analysis), and analyzing cytokine and chemokine patterns. Identifying specific biomarkers may help predict irAE development, stratify patient risk, and clarify the interplay between immune activation and antitumor responses.
- Genetic and genomic studies: Further investigation of genetic and genomic factors influencing irAE susceptibility and severity is essential. This includes conducting large-scale genome-wide association studies to identify novel genetic variants associated with hem-irAEs and evaluating their functional implications. Understanding genetic predisposition may enable personalized risk assessment and inform therapeutic decision-making.
- Clinical trials on immunosuppressive treatment strategies: Prospective clinical trials are needed to evaluate the optimal timing, dosage, and duration of immunosuppressive therapy for hem-irAEs. These trials should assess the impact of different management strategies on both irAE resolution and oncologic outcomes, with the aim of identifying approaches that effectively control toxicity without compromising antitumor efficacy.
- Mechanistic studies on immune cross-reactivity: Further mechanistic investigations are required to elucidate the precise molecular and cellular basis of immune cross-reactivity between tumor antigens and self-antigens in hematolymphoid malignancies. Such studies may involve advanced immunologic techniques to characterize shared epitopes and the corresponding T-cell and B-cell responses.
- Integration of multi-omics data: Integrating multi-omics data—including genomics, transcriptomics, proteomics, and metabolomics—from patients who experience hem-irAEs may provide a comprehensive view of the underlying biological pathways and identify novel therapeutic targets or prognostic markers.

By pursuing these future directions, researchers can build upon the existing knowledge base, refine understanding of hem-irAEs, and ultimately contribute to optimizing ICI therapy for patients with hematolymphoid malignancies.

## Conclusion

Hem-irAEs arising from ICI therapy are relatively rare but are increasingly recognized as potential indicators of enhanced immune activation associated with reduced relapse risk and improved survival. The complex interplay of T-cell–mediated cytotoxicity, B-cell dysregulation leading to autoantibody production, and generalized immune activation contributes to this phenomenon, often driven by immune cross-reactivity between tumor and self-antigens. The prognostic significance of hem-irAEs holds potential for clinical decision-making, offering insights into the efficacy of ICI therapy and informing patient management. However, the current evidence—largely derived from retrospective studies—underscores the need for prospective, controlled investigations to validate these associations and establish definitive guidelines for diagnosis and management. Future research efforts should focus on establishing standardized documentation, identifying robust biomarkers, elucidating genetic predispositions, and optimizing immunosuppressive strategies to balance toxicity control with antitumor efficacy.

**Fig. 1:**
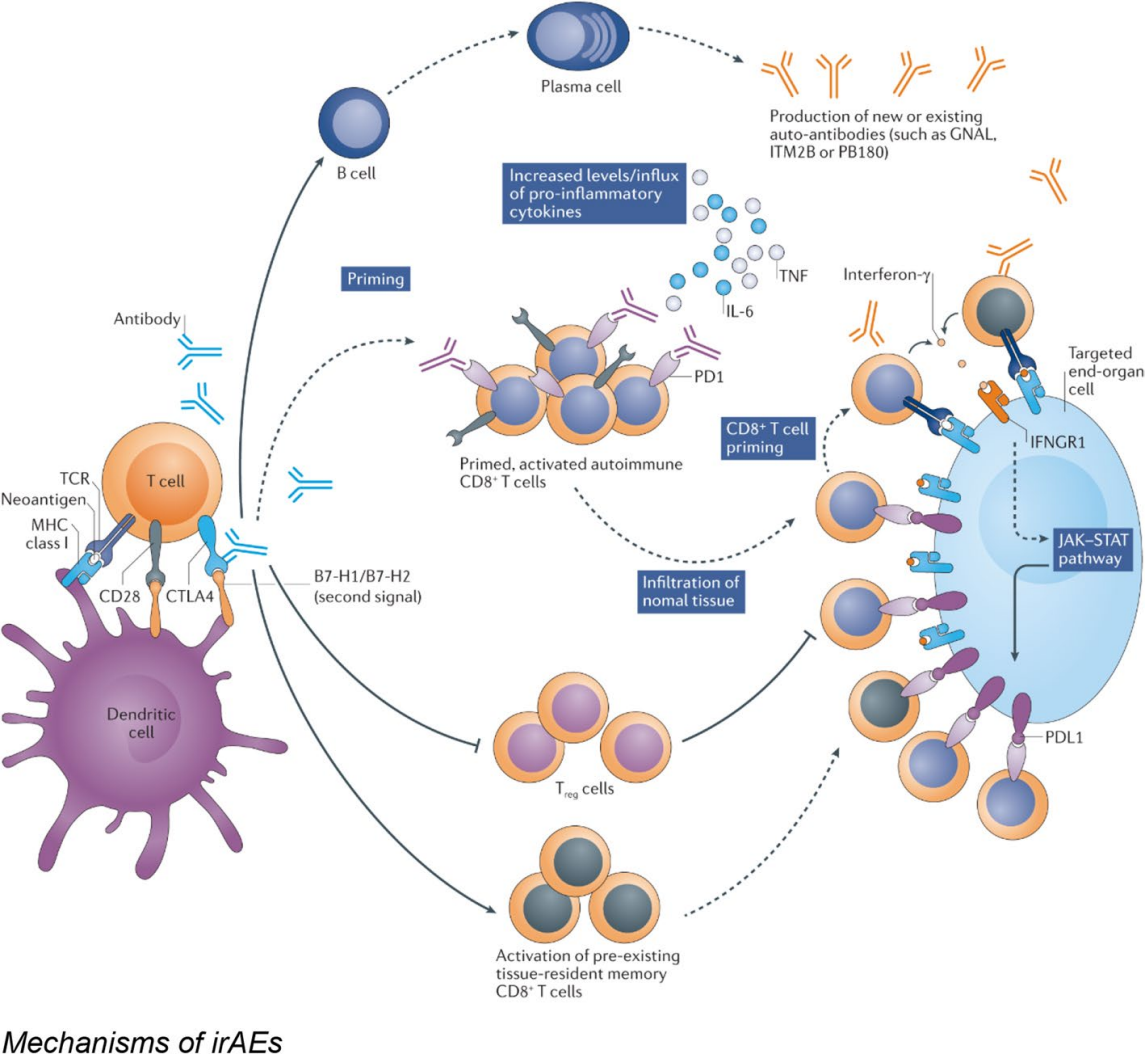
Proposed mechanisms of hematologic immune-related adverse events

Figure 1 illustrates the complex interplay of immune cells and molecular pathways leading to hematologic irAEs following immune checkpoint inhibition.

**Fig. 2:**
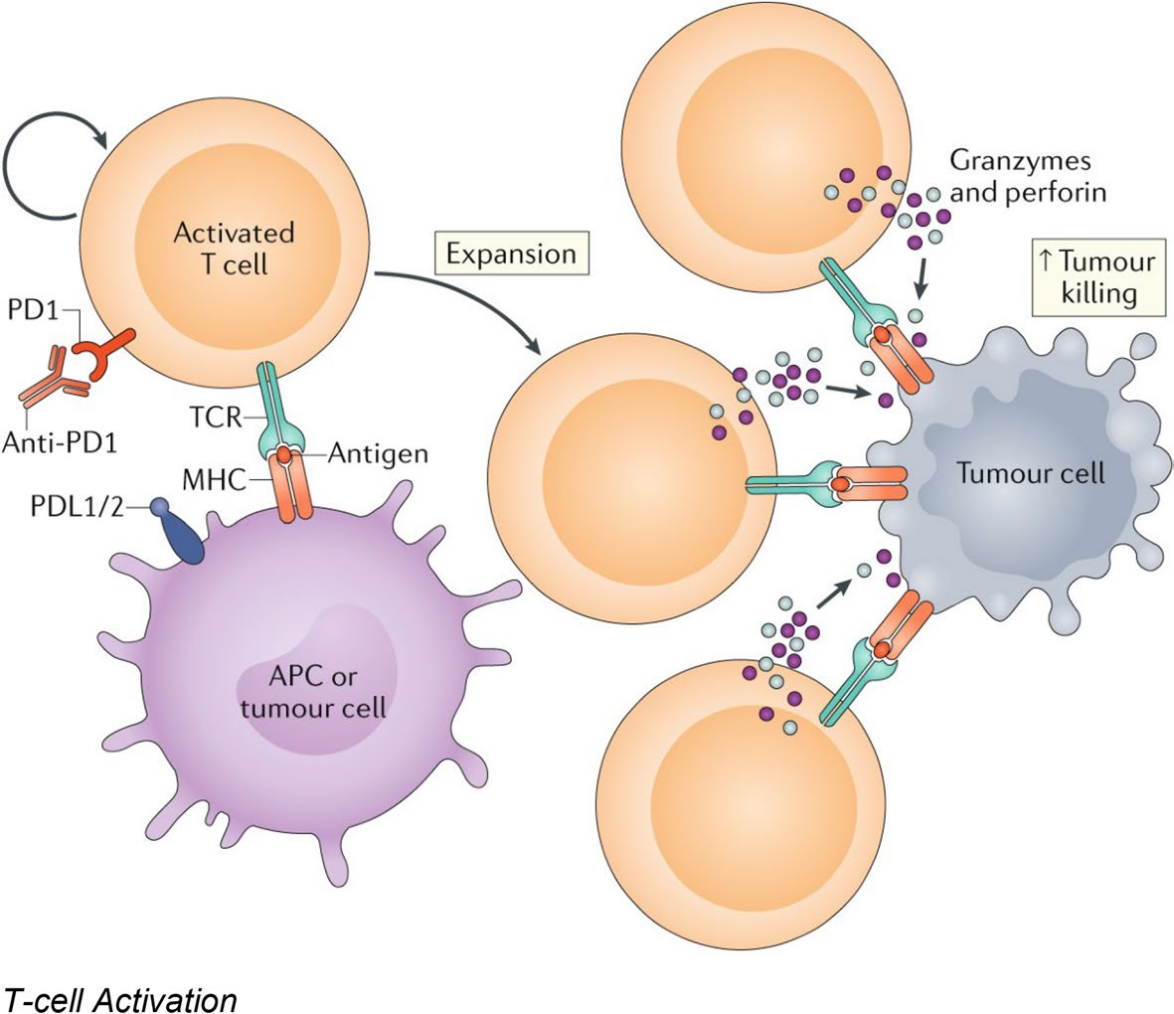
T-cell activation in cancer immunotherapy

Figure 2 depicts the process of T-cell activation in the context of cancer immunotherapy, highlighting the roles of antigen presentation and costimulatory signaling.

**Fig. 3:**
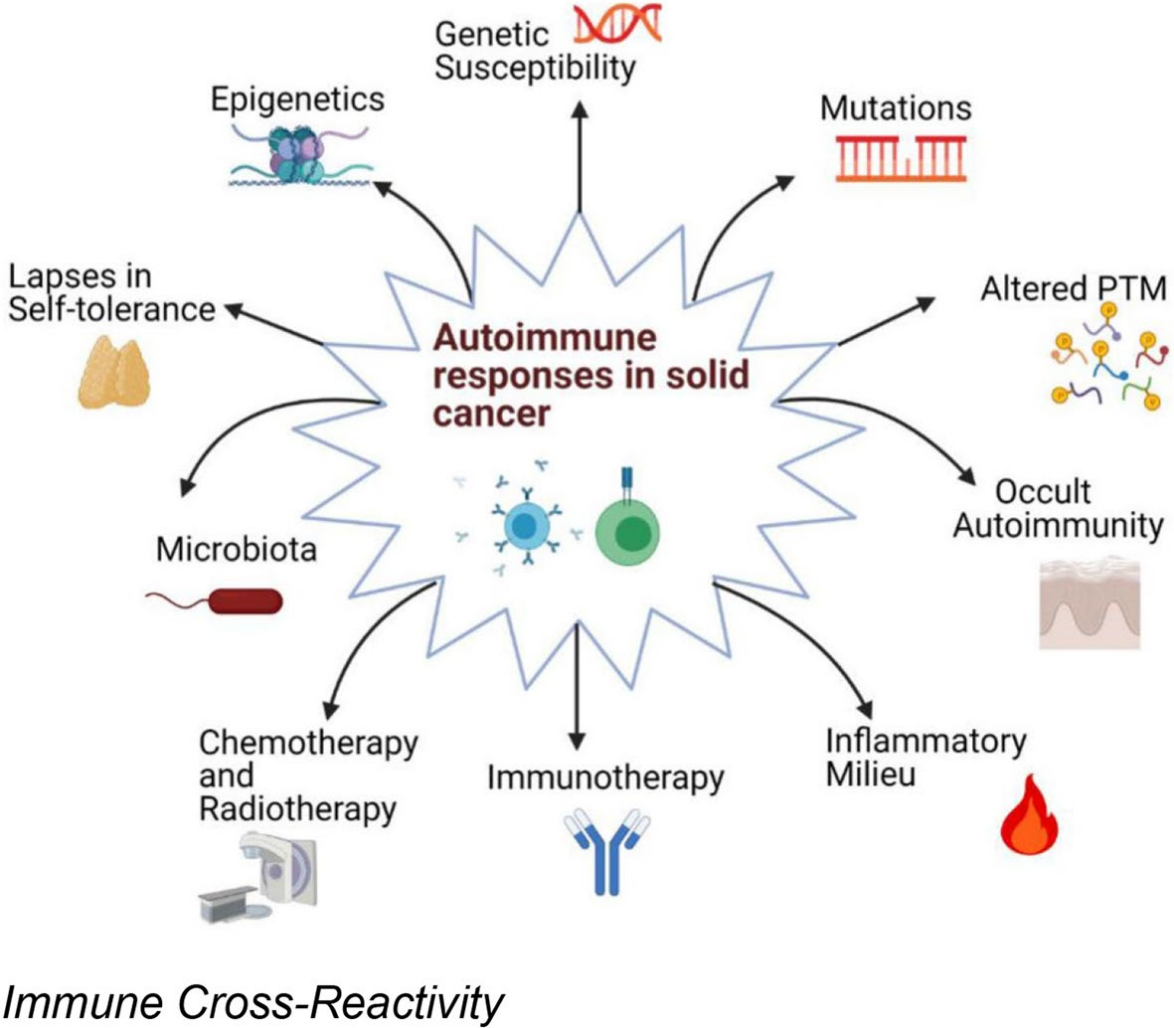
Immune cross-reactivity in autoimmune diseases and cancer

Figure 3 illustrates the concept of immune cross-reactivity, whereby immune responses directed against tumor antigens may inadvertently target self-antigens, resulting in autoimmune manifestations.

## Data Availability

No datasets were generated or analysed during the current study.
